# Severe Oxalosis With Systemic Manifestations

**DOI:** 10.4021/jocmr525w

**Published:** 2012-01-17

**Authors:** Majed Mark Samarneh, Norbert Shtaynberg, Michael Goldman, Edward Epstein, Morton Kleiner, Suzanne El-Sayegh

**Affiliations:** a Division of Nephrology, Department of Medicine, Staten Island University Hospital, Staten Island, NY 10305, USA

## Abstract

Ethylene glycol toxicity can have various clinical presentations with different organ system involvements. These presentations are independent of the level of toxicity. We describe a 31 years old male who presented with ethylene glycol toxicity manifesting as anuric renal failure who subsequently developed neurological sequela of its toxicity. Ethylene glycol is known to be metabolized to various metabolites and is ultimately converted to oxalate which results in crystal deposition the renal parenchyma causing renal failure. Oxalate deposition can occur in various organs including the nervous system as seen in our patient. The majority of patients do not recover from severe oxalosis despite the supportive care of hemodialysis in removing the parent compounds. Despite severe oxalosis, our patient was fortunate enough to be left with minimal neurological sequelae, and eventually was able to cease hemodialysis treatments.

**Keywords:**

Ethylene glycol; Oxalate; Oxalosis; Glyoxylic acid

## Introduction

Ethylene glycol intoxication can present in various ways. Identification and treatment of such intoxication is very important in order to avoid future complications. The metabolites of ethylene glycol are toxic and can result in multi-organ system damage. It is therefore important to understand the metabolism of the chemical and the pathophysiologic mechanisms by which it inflicts damage. Understanding these concepts will help one to readily identify the toxin, treat the patients, and manage the complications.

## Case Report

A 31 years old male with a past medical history of Hepatitis C, depression, and prior suicide attempts was brought to the emergency department (ED) by his family. Three days prior to admission he had ingested about 10 - 20 cc of antifreeze (ethylene glycol), 100 mg of Vistaril and 200 mg of Doxepinel in a suicide attempt. He had no contributory past surgical history or family history. He admitted to addiction to cocaine and heroin and was discharged from a detoxification facility 1 week prior to admission. He stated that he had no recollection of events that transpired between the ingestion of ethylene glycol and his presentation to the ED. He was found unresponsive at home and brought into the hospital and was awake and alert on presentation to the hospital.

In the ED the patient had a blood pressure of 103/59, heart rate of 92, respiratory rate of 18 and temperature of 100 degrees Fahrenheit. The patient was alert and oriented to self, location and time. The heart rate was regular with no murmurs, rubs or gallops, and the lungs were clear to auscultation, with no rales, rhonchi or wheezes. The abdomen was soft, non tender, not distended with no organomegaly, and bowel sounds were present in all four quadrants. The extremities had no edema, and had acceptable pulses were present. He was able to follow commands and did not have any short term memory defecit. Cranial nerves were intact, strength was 5/5 in all extremities, sensorium was intact to pinprick and light touch, and deep tendon reflex responses appropriate.

**Figure 1:**
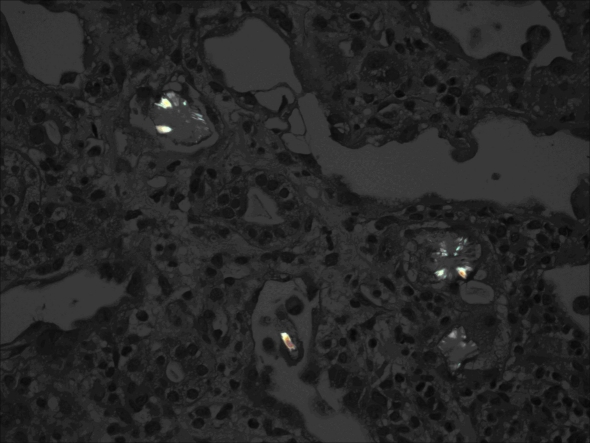
Renal core biopsy showed a significant amount of tubular injury caused by oxalate crystal deposition.

The patient was noted to have transaminitis which was attributed to Hepatitis C, and was also found to be in a high anion gap metabolic acidosis (17 mEq/L) with acute kidney injury (Creatinine 12.55 mg/dL), with total urine output of 5 mL during the first eight hours. Osmolar gap on presentation was within the normal range. He was admitted to the intensive care unit for treatment and observation and was started on intravenous normal saline, and oral pyridoxine and thiamine. An admission urinalysis had shown 3+ protein, 0 - 3 red blood cells, 6 - 12 white blood cells (per high power field), moderate bacteria, no casts, and no crystals. The fractional excretion of sodium was calculated to be approximately 20%. Renal sonogram revealed a right and left kidney of 13.4 and 14.2 cm respectively, with increased echotexture, and no hydronephrosis, masses or calculi. Serologic workup for causes of renal failure was found to be negative (C3, C4, antinuclear antibody, rheumatoid factor, anti-neutrophil cytoplasmic antibody and HIV). The cause of kidney injury was presumed to be due to oxalosis from the ethylene glycol ingestion. He continued to be anuric despite volume repletion and his electrolytes worsened, so he was started on hemodialysis. Renal core biopsy was obtained, and showed a significant amount of tubular injury caused by oxalate crystal deposition (Fig.1).

Five days after admission the patient began complaining of tinnitus in his right ear as well as vertigo and acute vestibular neuritis was suspected. MRI of the brain along with a lumbar puncture was performed. He was found to have sterile meningitis. He was started on steroids and meclizine without significant improvement in symptoms.

Twelve days after admission the patient began to complain of hearing loss in his right ear and developed left facial droop (distribution of V2 and V3) and right eye ptosis. His mental status began to worsen as he became increasingly lethargic, and he developed a fever and leukocytosis. He refused a repeat LP and empiric antibiotics were started. He began to have difficulty swallowing which was severe enough to require that all feeding be via a nasogastric tube. By this time, his hearing loss was profound, and it became difficult to communicate with him. Subsequently he began to have progressive shortness of breath and required intubation and mechanical ventilation. He failed to tolerate the removal of ventilatory support, and underwent tracheostomy and percutaneous endoscopic gastrostomy tube placement.

Three weeks after intubation, the patient began to regain consciousness and had progressive improvement of his mental status. Pt was successfully weaned off of the ventilator at this point. He began to open his eyes, then began to answer questions appropriately and progressively improved until he returned to the baseline mental status that he displayed upon arrival to the ED. He was discharged to an inpatient rehabilitation center where his condition continued to improve and he was able to fully ambulate after several weeks. He continued to require hemodialysis, but began to produce urine. Repeat microanalysis of his urine failed to indicate active sediment and his renal function did not improve. The patient was finally discharged home with outpatient hemodialysis three times a week. Approximately 5 months later, his renal function began to improve and hemodialysis was dicontinued. A partial facial palsy remains, as well as a mild hearing deficit.

## Discussion

Ethylene glycol, methanol, and isopropyl alcohol are toxic alcohols that are available in both commercial and household products and have been used in suicide attempts [1]. Methanol and ethylene glycol toxicity can be fatal and prompt intervention is warranted. Ethylene Glycol is a nonvolatile liquid that is colorless and odorless. It is found commonly in antifreeze, coolants, cleansers, and windshield de-icers [2]. Reporting of exposures to ethylene glycol is not mandatory so there may be an underestimation in national statistics. In 2007 the US poison centers received reports of 5731 cases that may have been exposed [3].

Historically, ethylene glycol toxicity has been divided into three stages and a proposed fourth stage, which will be discussed separately [4]. The first stage involves central nervous system depression and GI irritation. The initial symptoms may mimic those of ethanol intoxication but the patients don’t usually have the smell of alcohol on their breath unless there is co-ingestion. Gastritis may result from direct irritation of the gastrointestinal tract [4]. This stage may last from 30 minutes to 12 hours. The second stage of ethylene glycol intoxication involves cardiopulmonary collapse and occurs at about 12 to 24 hours after ingestion. The symptoms of this stage are tachypnea, hypotension, and congestive heart failure with pulmonary edema leading to acute respiratory distress syndrome [4]. Ethylene glycol is metabolized to its toxic compounds and anion gap metabolic acidosis occurs in this second stage [5]. The third stage of intoxication involves the kidneys and is termed the renal stage [1]. The metabolites of ethylene glycol are deposited in the kidney and the patient may develop an oliguric renal failure secondary to calcium oxalate crystal deposition [6]. The renal stage can last for months, as seen in our patient. The fourth stage of intoxication involves the neurologic complications that occur days to weeks after initial intoxication and may involve cranial nerves [7].

**Figure 2:**
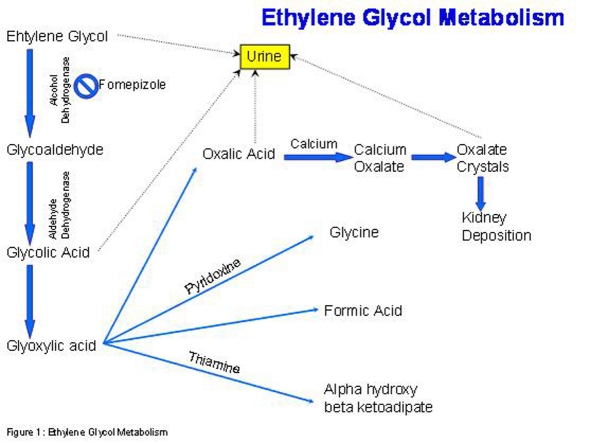
The metabolism of ethylene glycol.

The metabolism of ethylene glycol is of vital importance in understanding the serologic and clinical presentation (Fig. 2). Ehtylene glycol is metabolized by alcohol dehydrogenase (ADH) in the liver to which glycoaldehyde is in turn metabolized by aldehyde deyhdrogenase to glycolic acid [3,6]. Glycolic acid is metabolized by glycolate oxidase to glyoxylic acid which can now take one of several pathways. It can be converted by alanine-glyoxylate aminotransferase to glycine, which is a step that may be enhanced by administration of pyridoxine or be converted into formic acid and into alpha hydroxyl beta ketoadipate, the latter conversion enhanced by thiamine [1]. The fourth and most important pathway that glyoxylic acid can take is conversion to oxalic acid via the enzyme lactate dehydrogenase. Oxalic acid is excreted in the urine but when it binds calcium, it becomes calcium oxalate, which is the compound that forms oxalate crystals and gets deposited in the kidney causing kidney failure [1,6]. It is important to note here that hypocalcemia is among the electrolyte abnormalities that may develop as a result of this binding.

The recognition of ethylene glycol intoxication can be challenging. As discussed previously the initial presentation of altered mental status, nausea, and vomiting may be nonspecific and vague. It is therefore of critical importance to conduct an appropriate history from family or friends and a proper investigation for possible ingestion. Although our patient was awake when he presented to the ED, he was unable to recall the events that transpired from the time of consumption until the time of presentation. Direct ethylene glycol measurement is not available in many hospitals and the results do take some time to come back. Calcium oxalate crystalluria can be found in up to 50% of patients with ethylene glycol poisoning [1,8]. Crystals have been documented in urine for over a month after exposure to even modest amounts of ethylene glycol [8]. Another diagnostic clue is urine that is positive under a Wood’s lamp since fluorescein is added to antifreeze [1]. This is just a clue and a negative test should not be criteria for ruling out intoxication [1]. If the patient is anuric on presentation from kidney failure, it has been suggested to perform bladder irrigation with 100 mL of saline. The solution can then be prepared and examined for calcium oxalate crystals [9]. There have been cases of death reported with extremely high levels of ethylene glycol but without the crystalluria or the metabolites [10]. The patient discussed in this case had a tremendous amount of oxalate on kidney biopsy, but did not have any crystals in the urine.

The acid base presentation of ethylene glycol intoxication can vary depending on time after exposure [11]. The classic presentation is an osmolal gap which is followed by an anion gap metabolic acidosis. Ethylene glycol itself contributes to the osmolal gap, but this gap closes as ethylene glycol is metabolized [1]. The metabolites of ethylene glycol are what contribute to the anion gap. Out patient presented with an anion gap metabolic acidosis without an osmolal gap. This is likely due to metabolism of the parent alcohol, which contributes to the osmolal gap. Glycolic acid is the metabolite that contributes to the anion gap. This is important because the patient may present with osmolal gap alone initially, an osmolal and an anion gap metabolic acidosis, or with an anion gap metabolic acidosis alone [12]. There have also been a number of cases reviewed in the literature where patients present with non-anion gap metabolic acidosis [5]. In a number of these patients, no reason was identified for losses that would offset the anion gap. Other causes to consider of an anion gap metabolic acidosis include ketones, lactate, and organic acids from renal failure.

Criteria for initiating therapy for ethylene glycol include a plasma concentration of ethylene glycol of greater than 20 milligrams per deciliter, or a documented ingestion history with an osmolal gap of greater than 10 milliosmoles per liter, or suspected ingestion with three of the following parameters, arterial pH of less than 7.3, carbon dioxide of less than 20 mmoL per liter, an osmolal gap greater than 10 milliosmoles per liter or oxalate crystals in the urine [3].

Treatment for ethylene glycol intoxication involves the use of fomepizole (4-methylpyrazole) and hemodialysis. Since the metabolites of ethylene glycol are toxic substances, the enzyme alcohol dehydrogenase, which plays a role in this conversion, is the target of this drug. Intravenous ethanol has an affinity for alcohol dehydragenase that is 100 times more than ethylene glycol [1]. This relationship is important because patients who have co-ingestion of both ethylene glycol and ethanol will have a delayed presentation of ethylene glycol toxicity since it will take longer to metabolize the ethylene glycol. There are however side effects that come along with ethanol infusion including mental status changes, pancreatitis and hypoglycemia [1]. Fomepizole is a competitive inhibitor which reversible binds to alcohol dehydrogenase. Fomepizole does not have the side effect profile that ethanol has and its pharmacokinetics are more predictable [3]. Hemodialysis is used to remove the parent compound, ethylene glycol. Other agents that are given are pyridoxine and thiamine since they both help shift metabolism of glyoxylic acid to glycine and alpha hydroxyl beta ketoadipate, respectively [1]. The result is shifting the pathway away from formation of oxalate acid which when coupled with calcium is toxic to the kidneys and central nervous system.

As stated previously, calcium oxalate deposition in the kidneys causes toxicity and renal failure. Calcium oxalate monohydrate (COM) is the toxic agent [6]. COM is toxic to the proximal tubule, where it has been shown get internalized which results in cell death (acute tubular necrosis) [6]. There are several factors that can bind to COM and prevent them from binding membrane receptors. COM has a positively charged surface which reacts with several negatively charged sites on the cell membrane [6]. Polyanions that interact with the cell surface of COM crystals and inhibit membrane uptake include the glycoprotein osteopontin, glycosaminoglycans, and citrate [6]. COM crystals can also aggregate in the lumen of the tubule and this is inhibited by secretion of Tamm-Horsefall protein [6]. This differs from the pathologic mechanism of calcium oxalate nephrolithiasis in that kidney stones attach to Randall’s plaque in the papillary interstitium. Cell damage results from varying mechanism, not all fully understood, but involving mitochondrial dysfunction, membrane damage, and free radical generation [6]. When examined under polarized light, the crystals appear ”mussel-shaped“ [13]. Our patient had a tremendous amount of oxalate on kidney biopsy and required hemodialysis for approximately 5 months, after which he came off.

Ethylene glycol intoxication also has central nervous system manifestations which are described to be a stage in the intoxication cascade. A common finding involves central edema with basal ganglia and brainstem involvement [13]. The cortical gray matter is spared. Increased signal intensities can frequently be seen on MRI images of the basal ganglia [13,14]. Calcium oxalate crystals have also been found to be deposited in the walls of central nervous system blood vessels [15]. Autopsy findings include birefringent crystals in vessel walls and perivascular edema with polymorphonuclear leukocytes [15]. These findings are important because development of medications that inhibit the uptake of calcium oxalate monohydrate crystals may minimize or even prevent toxicity. Cranial nerve involvement is a common finding in the neurologic sequelae. The seventh and eight cranial nerves are commonly involved and it has been suggested to call this phase ”facial-auditory nerve oxalosis“ [16,17]. There have also been documented cases of deficits in other cranial nerves such as VI, IX, and X which result in visual disturbances and dysphagia [18,19]. Our patient also displayed a neurological sequel which involved several cranial nerves similar to the case reports described. Ethylene glycol intoxication can also result in a neuropsychological sequelae [20]. Patients may have a decline in cognitive function that persists months after intoxication [20]. Patients may also have meningismus. Deposition of calcium oxalate in the meninges may result in a sterile meningitis [21]. A lumbar puncture performed on our patient also revealed a sterile meningitis. Cerebrospinal fluid may show elevated protein or polymorphonuclear pleocytosis [21].
